# The role of day-case thoracoscopy at a district general hospital: A real world observational study

**DOI:** 10.1016/j.fhj.2024.100158

**Published:** 2024-07-04

**Authors:** Sidra Kiran, Akash Mavilakandy, Sarah Rahim, Muhammed Naeem, Samantha Rawson, Darren Reed, Georgios Tsaknis, Raja V. Reddy

**Affiliations:** a Department of Respiratory Medicine, Kettering General Hospital, Kettering General Hospital NHS Trust, UK; b Department of General Medicine, Leicester Royal Infirmary, University Hospitals of Leicester, UK; c Department of Respiratory Medicine, Northampton General Hospital, Northampton General Hospital NHS Trust, UK; d Department of Respiratory Sciences, University of Leicester, Leicester, UK

**Keywords:** Medical thoracoscopy, Local anaesthetic thoracoscopy, Day-case, Talc pleurodesis, Talc poudrage, Indwelling pleural catheter, IPC

## Abstract

**Objective:**

To assess the feasibility and safety of talc pleurodesis performed as part of day-case medical thoracoscopy.

**Methods:**

A Richard Wolf® 5 mm mini thoracoscope through a 5.5 mm port was used with eligible cases having talc poudrage followed by insertion of indwelling pleural catheter (IPC). District nurses drain the IPC daily for the first 5 days. Once the drain output is <150 mL, the frequency is progressively reduced to once weekly. The drain is removed after two consecutive dry taps 1 week apart.

**Results:**

Overall, 51 patients underwent day-case thoracoscopy. Median time to removal of IPC for our day-case protocol was 14 days. There were seven deaths within 70 days among 41 patients with malignant pleural effusion in the day-case cohort, compared to eight deaths in the 33 conventional thoracoscopy controls. Overall, the day-case cohort observed a statistically significant reduction in all-cause mortality at 180 days compared to the conventional cohort (log rank *p =* 0.024). The average cost per patient of the day-case and inpatient cohort was £1,328.0 ± 106.0 and £1,835.0 ± 295.0 (*p =* 0.961).

**Conclusion:**

This study suggests that thoracoscopy and talc poudrage can be performed safely as a day-case procedure. Further data are needed to ascertain long-term outcomes.

**What is already known on this topic** – Medical thoracoscopy in combination with talc pleurodesis is commonly used for management of malignant pleural effusion. The procedure conventionally necessitates a subsequent inpatient admission.

**What this study adds** – This study has added evidence to the feasibility, efficacy and cost-effectiveness of medical thoracoscopy with talc pleurodesis as a day-case elective procedure.

**How this study might affect research, practice or policy** – This study may encourage further larger and robust randomised control trials to contribute to the evidence base surrounding this intervention. If the findings are consistent across studies, day-case implementation could be highly beneficial to the patient as well as the healthcare infrastructure.

Alt-text: Unlabelled box

## Introduction

Shortness of breath due to pleural effusion is a highly common presenting complaint and indication for hospital review and admission. Pleural effusion is an excessive accumulation of fluid in the pleural space secondary to imbalance of pleural fluid formation and removal, which is frequently encountered in clinical practice.^1^ There is a myriad of pathologies that can cause an effusion, with heart failure, neoplastic and infective aetiologies as the top three precipitants.^2^ Malignant pleural effusion (MPE) is associated with increased morbidity and mortality and constitutes a significant proportion of hospital admissions.^3^ The conventional treatment approach is centred around symptomatic management with a particular emphasis on relief of dyspnoea, discomfort and hospitalisation avoidance.^4^, ^5^, ^6^ Serial monitoring and outpatient observation can be used for asymptomatic patients, while several therapeutic approaches can be employed dependent on clinical factors such as performance status, primary disease prognosis, as well as logistical variables including accessibility to thoracoscopy.

Medical thoracoscopy is a procedure performed under conscious sedation and local anaesthetic, with trocars inserted in the intercostal space to allow direct visualisation of the pleural space and facilitation of fluid drainage, tissue biopsies, as well as administration of a sclerosing agent such as talc.^7^ Pleurodesis is a procedure facilitated to obliterate the pleural space through mechanical or chemical processes to prevent recurrent pleural effusions or pneumothoraxes.^8^ Conventionally, medical thoracoscopy when combined with pleurodesis is associated with prolonged hospital admissions ranging from 4.5–6 days.^9^, ^10^, ^11^ Attempts of facilitation as a day case in the absence of pleurodesis have been successful. There is, however, a paucity of data regarding feasibility of pleurodesis in addition to medical thoracoscopy as a day case and such no protocol has been synthesised or reported in literature thus far.

This study reports the findings of a large district general hospital's experience in execution and feasibility of medical thoracoscopy with pleurodesis as an elective day-case procedure.

## Methodology

### Approval and setting

The study was a service evaluation of pleural effusion management as an elective/outpatient service at a secondary care centre, which serves as a referral centre for complex pleural disease case management within a large county. The study was presented and discussed with the local institution research ethics committee, who confirmed that research committee approval was not required for the conduction of this study as it was a retrospective comparison of the institution's standard practice.

The study was conducted as a single-centre, retrospective observational study that compared two independent cohorts of patients. The first cohort was composed of patients who underwent local anaesthetic video assisted thoracoscopy (LAVAT) and pleurodesis through the ambulatory day-case pathway from April 2019 to October 2020. All elective patients were consecutively listed and included in the study. The second cohort is derived from retrospective data of patients who underwent conventional LAVAT with subsequent inpatient stay from April 2017 to November 2017, prior to the introduction of the day-case thoracoscopy ambulatory pathway. Both cohorts omitted medical thoracoscopy cases performed for inpatients who were admitted for reasons other than procedure facilitation.

### Inclusion and exclusion criteria

The inclusion criteria were:

1. Undiagnosed recurrent exudative pleural effusions
2. Recurrent malignant pleural effusion requiring pleurodesis in patients with a WHO (World Health Organization) performance score (PS) / Eastern Cooperative Oncology Group (ECOG) of 2 or less (Supplementary file 1)^12^
3. Elective admissions only

The exclusion criteria were:

1. Patients demonstrating absolute contraindications to LAVAT based on the current British Thoracic Society (BTS) guidelines (Supplementary file 2).^13^
2. Presence of 'trapped lung’ diagnosed prior to procedure. A trapped lung is defined as the inability of lung to expand and fill the thoracic cavity as a result of a fibrinous restrictive pleural layer than impedes normal visceral and parietal pleural apposition.^14^
3. Life expectancy of <3 months.

### Daycase LAVAT protocol

**Fig. 1:**
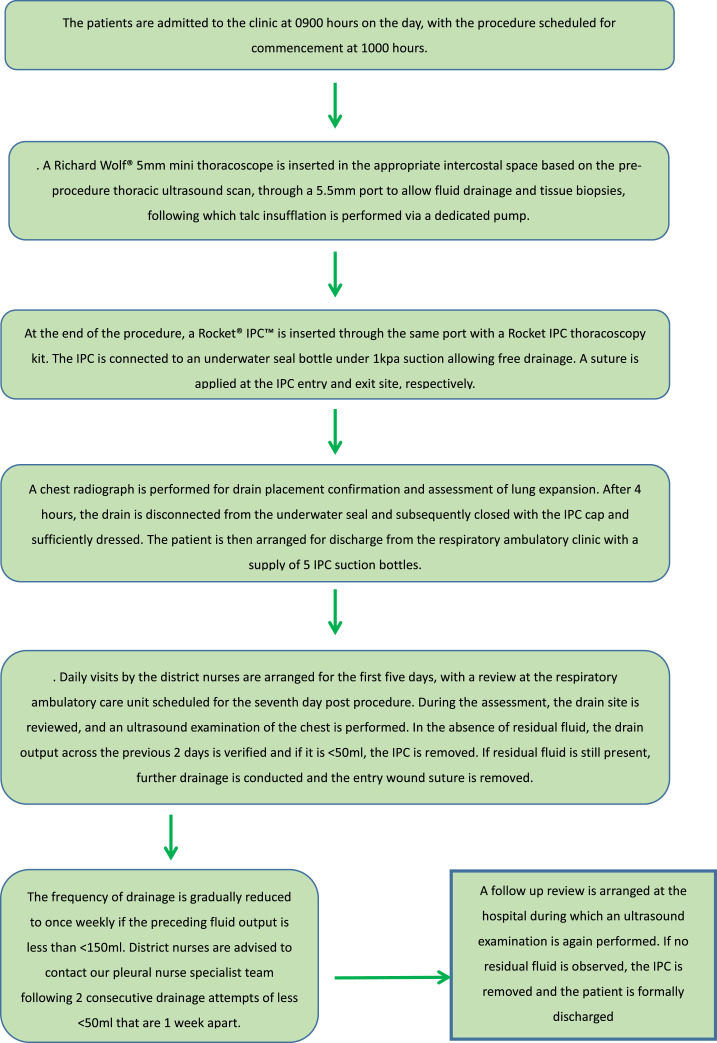
Daycase local anaesthetic video-assisted thoracoscopy protocol.

Patients referred for LAVAT were first assessed in the respiratory ambulatory clinic prior to the procedure; those requiring pleurodesis as well as conforming to the inclusion criteria were included in the study. Fig. 1 illustrates the day-case LAVAT protocol. Any unarranged/unscheduled use of healthcare resources in the community is queried by reviewing local database records, while any events of hospitalisation are tracked on Kettering General Hospital and Northampton General Hospital's Patient Administration Systems (PAS).

With respect to the retrospective comparator cohort, patients had a size 24 French drain inserted through the same port at the end of the procedure and were admitted to hospital. The chest drain was removed once the drainage volume was observed to have been less than 200 mL/day.

### Demographics

Demographic information was collected corresponding to age, gender, site of effusion, WHO performance score, comorbidities, malignant primary source of effusion, and LENT score was calculated. The LENT score is a validated risk stratification system used to predict the survival in MPE on the basis of variables including performance status, tumour type, serum neutrophil-to-lymphocyte ratio, and pleural fluid lactate dehydrogenase levels.^15^

### Outcome

The primary outcome was the number of patients who met the criteria for drain removal within 70 days of insertion. A 70-day cut-off was used to enable comparison with published data as previous major studies utilised this time-point for assessing outcomes.^16^

Secondary outcomes were:

- Total number of days of all-cause hospitalisation within 70 days post-procedure
- Total number of patients requiring a repeat procedure for recurrence of pleural effusion within 70 days post-procedure
- Total number of complications, ie drain displacement / accidental removal, pleural infection
- Total number of patients with unexpanded lung on the post-procedural chest radiograph
- Total number of patients with subcutaneous emphysema immediately post-procedure
- Total number of patients with unexpected healthcare utilisation in the 70 days post-procedure
- Incidence of all-cause mortality within time points of 60 days and 180 days
- Financial cost involved in facilitation of the pathways – The total cost includes the professional involvement for each post-procedural visit and all consumables required.

### Focused literature review

The following databases of Medline, Embase and Cochrane were searched by AM for studies reporting outcomes on daycase medical thoracoscopy with IPC insertion that were published up to 1 October 2023. The specific search strategy is available in Supplementary Digital File 3.

### Statistical methods

Categorical variables were expressed as frequency and percentage. Median and interquartile range (25th-75th percentiles) were used to describe non-parametric continuous data. Mean and corresponding confidence intervals were used to describe parametric continuous data.

Comparison between unpaired groups of parametric data was conducted with the Student's *t*-test while non-parametric data unpaired comparisons were conducted with the Mann–Whitney U test. Categorical variables were compared with the use of the χ^2^ test. A *p* < 0.05 was considered statistically significant.

Univariate and multivariate Cox proportional hazard regression analyses were carried out to identify factors having a significant impact on overall survival within 180 days. Significant variables (*p* < 0.05) in the univariate Cox proportional hazard regression analysis were entered into the multivariate analysis. The associated results were reported as hazard ratios (HRs) with 95% confidence intervals. Kaplan–Meier curves were used to depict survival distribution from all-cause mortality between the day-case and inpatient cohort, while a log-rank test was used to compare overall survival between the two groups. Binary logistic regression was performed to identify factors having a significant impact on drain removal within 70 days of procedure.

Statistical analysis was conducted with the use of GraphPad Prism 8 (GraphPad Software, Inc, CA, USA) and IBM SPSS Statistics Version 28.0.0.0 (IBM, NY, USA).

## Results

From April 2019 to October 2020, 51 patients had medical thoracoscopy with pleurodesis performed as a day-case procedure vs. 37 patients in the comparator cohort that had medical thoracoscopy with pleurodesis performed with planned inpatient stay from April 2017 to November 2017. The clinical characteristics of the patients are included in Table 1*.* The median age (IQR) of the day-case cohort was 71 (60–78) years vs. 70 (60.5–78) years in the inpatient group. Approximately 65% of the day-case group were male in comparison to 76% of the inpatient cohort. The median WHO performance score for the day-case group was 1 (0–1) vs 1 (0–2) in the inpatient group. The median Charlson comorbidity index score for the day-case group was 8 (5–11) vs. 6 (4–9) in the inpatient group. Malignant aetiologies were responsible for 76.5% of pleural effusions in the day-case group vs 89.2% in the inpatient cohort. Mesothelioma was the most frequently observed malignancy in both cohorts, with 17 (33.3%) cases in the day-case cohort vs. 16 (43.2%) cases in the inpatient cohort (*p =* 0.343). The median LENT score was 2 (1–3) for the day-case group vs 2 (0–4) in the inpatient cohort. Benign aetiologies were associated with 10 (19.6%) of pleural effusions in the day-case cohort vs. 4 (7.84) pleural effusions in the inpatient cohort, with non-specific inflammatory pathologies responsible for majority of cases across both cohorts.

**Table 1:** Comparison of baseline characteristics.

Patient demographics	Day case (*n =* 51)	Conventional (*n =* 37)	*P* value
Gender			
Male (%)	64.7	75.7	0.271
Age/years (median (IQR))	71 (60–78)	70 (60.5–78)	0.987
WHO PS (IQR)	1 (1–4)	1 (0–2)	0.208
Charlson comorbidity index (IQR)	8 (5–11)	6 (4–9)	0.107
Lactate dehydrogenase (IQR)	236 (163.5–449.5)	297 (194–503)	0.478
**Aetiology of pleural effusion**			
Malignancy (%)	39 (76.5)	33 (89.2)	0.127
Pulmonary (%)	12 (23.5)	6 (16.2)	0.401
Adenocarcinoma (%)	9 (17.6)	4 (7.84)	0.372
Non-small cell lung carcinoma (%)	1 (1.96)	1 (2.7)	0.818
Squamous cell (%)	0 (0)	1 (2.7)	0.238
Non specified (%)	2 (3.92)	0 (0)	0.223
Mesothelioma (%)	17 (33.3)	16 (43.2)	0.343
Mesothelioma epithelioid (%)	10 (19.6)	8 (21.6)	0.817
Mesothelioma unspecified (%)	7 (13.7)	8 (21.6)	0.331
Other primary (%)	10 (19.6)	11 (29.7)	0.272
Breast (%)	5 (9.80)	2 (5.41)	0.452
Ovarian (%)	2 (3.92)	3 (8.11)	0.402
Cholangiocarcinoma (%)	1 (1.96)	0 (0)	0.392
Gastrointestinal (%)	1 (1.96)	3 (8.11)	0.172
Lymphoma (%)	0 (0)	2 (5.41)	0.093
Neuroendocrine (%)	1 (1.96)	0 (0)	0.392
**LENT Prognostic score for malignant pleural effusion (IQR)**	2 (1–3)	2 (0–4)	0.804
Benign (%)	12 (23.5)	4 (7.84)	0.127

IQR *Interquartile range;* WHO PS *World Health Organi*z*ation Performance Status; LENT prognostic score for MPE*.

*P*-values were generated the Mann–Whitney test or χ^2^ testing depending on the type of variable.

### Outcomes

Of 51 patients in the day-case cohort, 50 successfully underwent the procedure while one patient required a 1-day planned admission in view of considerations towards a past medical history of learning disabilities. In comparison, all 37 patients were successfully facilitated for medical thoracoscopy and talc pleurodesis in the inpatient comparator group (Table 1).

With respect to the primary outcome, 42 (82.3%) patients in the day-case cohort had their drains successfully removed within 70 days vs. 37 (100%) patients in the inpatient group (*p =* 0.007). The median drain *in situ* duration was 14 days (9–30.5) for the day-case group vs. 3 days (2–7) for the inpatient group (*p* < 0.0001) (Table 2).

**Table 2:** Subacute/long term outcomes.

	Day-case (*n =* 51) (%)	Conventional (*n =* 37) (%)	*P* value
Number of patients requiring hospitalisations within 70 days	13 (25.5)	11 (29.7)	0.659
Total number of hospitalisations within 70 days	16	15	N/A
Total number of inpatient bed-days	79	78	0.0845
Repeat pleural procedure within 70 days	1 (1.96)	5 (13.5)	0.0338
All-cause mortality at 60 days	4 (7.84)	6 (16.2)	0.222
All-cause mortality at 180 days	11 (21.6)	17 (46.0)	0.0154
All-cause mortality at 365 days	22 (43.1)	23 (62.2)	0.0780
All-cause mortality at 60 days – MPE	4 (10.3)	6 (18.2)	0.333
All-cause mortality at 180 days – MPE	10 (25.6)	15 (45.5)	0.0785
All-cause mortality at 365 days – MPE	19 (48.7)	21 (63.6)	0.204
Total cost across cohort/£	£ 66,380.0	£67,900.0	N/A
Average cost per patient +/- SEM/£	£1,328.0 ± 106	£1,835.0 ± 295	0.961

MPE *Malignant pleural effusion*; SEM *Standard error of mean*.

A total of 13 (25.5%) in the day-case group vs. 11 (29.7%) patients in the inpatient group required hospital acute admission within 70 days of drain insertion (*p =* 0.659). A total of 16 acute hospital admissions within 70 days of drain insertion were observed in the day-case group vs. 15 admissions in the inpatient group (*p =* 0.641) (Table 2). The total number of inpatient hospitalisation days after the procedure was 79 in the day-case cohort vs. 78 in the inpatient cohort (*p =* 0.0845). The average duration of subsequent inpatient hospitalisation for the daycase cohort was 3 (1.5–10) days vs 22.5 (5.5–30.5) days for the inpatient cohort (*p =* 0.0845). Reasons for acute admissions of the day-case cohort included community-acquired pneumonia, PE and COVID-19 pneumonitis, while some admissions were for non-respiratory-associated pathologies (multifactorial falls, large bowel obstruction, blocked catheter). Within the day-case cohort, one (1.96%) patient required a repeat pleural procedure; a therapeutic aspiration performed approximately 1.5 months following drain removal. In comparison, the inpatient cohort observed five (13.5%) repeat procedures; three therapeutic pleural aspirations, one indwelling pleural catheter insertion and one referral to the thoracic surgical team for evacuation and biopsy (*p =* 0.0338) (Table 2). There was no recorded or documented procedural complications or adverse events on either treatment groups. Regarding lung expansion, 32 patients in the day-case cohort vs. 28 patients in the inpatient cohort demonstrated full lung expansion at the time of discharge from hospital (*p =* 0.199). A total of 14 (27.5%) patients from the day-case cohort vs. 11 (29.7%) from the inpatient cohort demonstrated surgical emphysema post-procedure, respectively (*p =* 0.815).

**Fig. 2:**
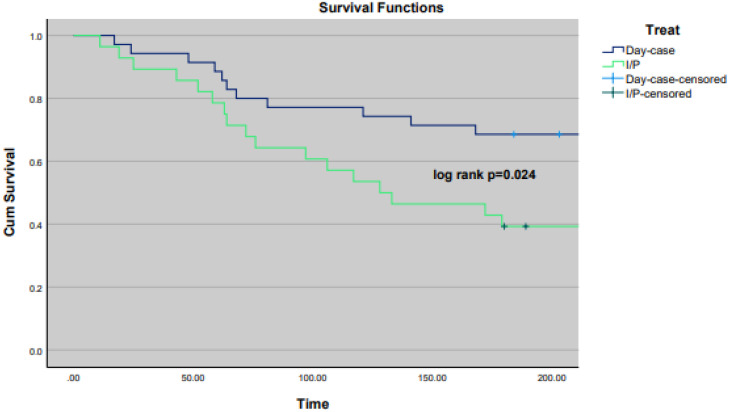
Survival analysis – Kaplan–Meier – mortality at 180 days.

For all-cause mortality at a 60-day time point, four (7.84%) events were recorded in the day-case cohort vs. six (16.2%) events in the inpatient cohort (*p =* 0.222). Regarding 180-day mortality, 11 (21.6%) events were observed in the day-case cohort vs 17 (46.0%) events in the inpatient cohort (log rank *p =* 0.024) (Fig. 2). In addition, 180-day all-cause mortality for MPE was 10 (25.6%) in the day-case group vs. 15 (45.5%) in the inpatient cohort (*p =* 0.0785).

Binary logistic regression did not identify any statistically significant associations between explored factors and drain removal within 70 days (Supplementary Table 1). The results of the Cox proportional hazards regression analysis of potential factors affecting overall survival are shown in Supplementary Table 2. Univariate analysis did not identify any statistically significant associations between explored factors and change in overall survival. In view of the absence of statistically significant associations, multivariate analysis was not pursued.

The total cost of the day-case cohort (*n =* 51) was £66,380.00 vs. £67,900.00 for the inpatient cohort (*n =* 37). The corresponding average cost per patient of the day-case cohort was £1,328.0 ± 106.0 vs. £1,835.0 ± 295.0 for the inpatient cohort (*p =* 0.961) (Table 2).

## Discussion

In 2019, The European Respiratory Society (ERS) and European Association for Cardio-Thoracic Surgery (EACTS) established guidance and recommendations on the use of talc pleurodesis or IPC in the management of MPE.^17^ With respect to modalities of talc pleurodesis, the TAPPS trial conveyed a similar efficacy in pleurodesis success rate for talc poudrage in comparison to talc slurry, although the required length of admission for facilitation was less than enviable (5–6 days).^11^ On the other hand, IPC has an established role in MPE with particular benefit noted in symptom alleviation, but a comparatively reduced performance profile in auto-pleurodesis even when facilitated with adjunctive therapies of talc administration^18^ or aggressive drainage.^19^ Furthermore, the additional caveat of an IPC-centric approach also involved logistical feasibility of drainage frequencies, susceptibility to IPC-associated infections, as well as cost of equipment such as drainage bottles. As a result, there has been increasing interest surrounding the feasibility of day-case thoracoscopic pleurodesis with IPC insertion. From this population's perspective, especially when considering the potentially limited median survival, optimising time spent in their preferred setting can be among the greatest of priorities for both the patient and their loved ones, but also the healthcare professionals. Furthermore, in recent times following the COVID-19 pandemic, bed capacity has been a ubiquitous challenge encountered by healthcare organisations and thus a safe and reliable adaptation of services to an ambulatory format would be highly desirable. Aggressive drainage strategies can incur a significant logistical strain and is particularly contingent on the healthcare infrastructure of the institution. This strategy has, however, been demonstrated to increase the probability of pleurodesis in several trials including the IPC-Plus, AMPLE-2 and SAP trial.^18^, ^19^, ^20^

The findings from our study demonstrate feasibility and non-inferiority of day-case medical thoracoscopy with pleurodesis and aggressive drainage via IPC, in comparison to the conventional inpatient approach. All patients were successfully discharged on the same day of the procedure, barring one patient who was already planned for a 1-day admission for facilitation of the procedure in consideration of learning disabilities. Just above 80% of patients in the day-case cohort successfully achieved the primary outcome of planned drain removal within 70 days following successful pleurodesis, with a median *in situ* duration of 14 days in comparison to 3 days for the inpatient cohort. This discrepancy in duration was entirely expected as the comparator arm had drainage performed exclusively with a large bore intercostal drain during their inpatient admission.^21^ Physicians involved in the facilitation of the day-case pathway reported feasibility and sustainability, with the pathway now utilised at present as the preferred approach for this specified indication at the institution. Physicians have also conveyed logistical benefit as the previous pathway requiring inpatient stay often observed cancellations and rescheduling because of challenges with bed capacity. Previous data also conveyed patient preference against inpatient admission following medical thoracoscopy.

A focused literature search retrieved a total of 49 studies. Following title and abstract screening, four studies were included,^22^, ^23^, ^24^, ^25^ all of which were retrospective observational in methodology. The extracted data and outcomes can be reviewed in Table 3. The findings of this study's day-case cohort were better than what was achieved in a UK-based case series reported by Foo *et al.* that reported outcomes following ambulatory thoracoscopic pleurodesis in conjunction with an IPC and less than aggressive drainage for management of MPE.^25^ The authors reported a 77.8% successful pleurodesis rate at 6 months follow-up, a median length of stay (LOS) of 0 (0–0) days and a median IPC *in situ* duration of 20 days. Turner *et al* conducted a similar study and reported two-centre findings corresponding to the day-case local anaesthetic thoracoscopy (LAT) with IPC insertion and reaffirmed the feasibility of this approach. However, it is important to mention that their protocol did not include talc pleurodesis / poudrage and an aggressive drainage approach due to logistical constraints.^23^ There are currently no published randomised controlled trials (RCT) evaluating day-case thoracoscopy with IPC insertion; however, the ongoing Randomised Thoracoscopic Talc Poudrage + Indwelling Pleural Catheters versus Thoracoscopic Talc Poudrage only in Malignant Pleural Effusion (R-TACTIC) trial has completed recruitment and randomisation of patients into treatment arms of thoracoscopy with talc and IPC, or to receive thoracoscopy and talc alone.^26^ This RCT similarly employs an aggressive drainage strategy with primary outcomes particularly focused on qualitative assessment of breathlessness as well the number of days spent in hospital. In addition to the outcomes presented by the literature, our study presented findings corroborating to readmission rates, all-cause mortality at particular time points, as well as a health-economic cost analysis. Interestingly, the day-case population demonstrated a statistically significant reduction in mortality at 180 days in comparison to the inpatient population. It is, however, important to note that when examining 180-day mortality for specifically MPE, there was a non-statistically significant reduction (25.6% vs. 45.5%, *p =* 0.0785). The inpatient cohort also demonstrated a statistically significant increase in repeat procedures within the 70-day interval. The two respective cohorts did not differentiate significantly in view of age, aetiology or LENT score, or WHO PS. Furthermore, there were no post-procedural adverse events or complications although, in the context of post procedural adverse events, it is important to note that the study's sample size is not sufficiently powered to accurately depict complications rate. Lastly, number of observed hospital admissions post-procedure was similar and comparable across both cohorts.

**Table 3:** Focussd literature review.

Study	Country	Type of study	Sample size	Day-case LA thoracoscopy	Talc pleurodesis/poudrage	IPC insertion	IPC/procedural complication rate (%)	Same day discharge (%)	Median Duration of IPC/days (IQR)	Successful pleurodesis (%)
Psallidas *et al.*	United Kingdom	Retrospective observational	242	✓	×	×	1.7	100	N/A	N/A
Turner *et al.*	United Kingdom	Retrospective observational study	79	✓	×	✓	9	88.0	*Almost 80 days	N/A
Kyskan *et al.*	Canada	Retrospective cohort study	218	✓	×	✓	6.5	99.1	⁎⁎34 (14.0–81.5)	N/A
Foo *et al.*	United Kingdom	Retrospective chart-based study	45	✓	✓	✓	0	86.7	⁎⁎20 (13–48)	3 months – 71.1% 6 months – 77.8%

LA *Local anaesthetic*; IPC *Indwelling pleural catheter*; N/A *Not applicable/available*.

⁎⁎ Median and associated interquartile range.

As a result, the discrepancy in all-cause mortality could be potentially explained by increased risk of hospital-acquired pathologies such as infections^27^ with inpatient stay along with hospital-associated deconditioning.^28^ Furthermore, it is also important to consider the effect of systemic cancer therapy on survival. A significant proportion of patients were on chemotherapy and, with newer agents such as immunotherapy introduced over time, there is a potential this could have also contributed to the improved survival observed in the day-case cohort. Overall, the reduced mortality and absence of pleural-related complications is reassuring and does not lend support to the theoretically postulated increased risk of infections secondary to frequent drain manipulation as part of the aggressive drainage protocol.

An attempt was made to identify predictors of poor outcomes corresponding to unsuccessful pleurodesis and mortality in the daycase cohort to aid stratification and patient selection for this approach. Binary logistic regression was performed and did not identify any particular variables corresponding to failed pleurodesis or in this study's context, drain *in situ* duration for more than 70 days. Similarly, Cox proportional hazard regression analysis was unable to identify any predictors of mortality in the day-case cohort.

Our study has multiple limitations. Firstly, this is a non-randomised retrospective observational study with a non-matched control group for comparison. Furthermore, a formal power calculation was not pursued in determining a sample size and thus the study was susceptible to type 2 random error. Thirdly, there was no formal evaluation into patient-related outcomes measures such as subjective improvement in dyspnoea and quality of life, which may have added more information to the efficacy of this intervention.

## Conclusion

In spite of the limitations, our study has demonstrated the feasibility and efficacy of a day-case medical thoracoscopy with talc poudrage and IPC insertion with respect to clinical and health-economic outcomes. The findings regarding patient-related outcome measures from the R-TACTIC trial will be highly awaited and contribute greatly to the management of MPE.

## Declaration of competing interest

The authors declare that they have no known competing financial interests or personal relationships that could have appeared to influence the work reported in this paper.

## Appendix Supplementary materials

Image, application 1

Image, application 2

Image, application 3
