# Supplementary material for: The role of day-case thoracoscopy at a district general hospital: A real world observational study
Source: Future Healthc J. 2024 Jul 4;11(3):100158. doi: 10.1016/j.fhj.2024.100158 (PMC11357848; doi:10.1016/j.fhj.2024.100158)
Supplement: Supplementary file 1 [file mmc1.docx]

**Supplementary File 1.**

| WHO PS/ECOG Score(1) | Patient status |
| --- | --- |
| 0 | Able to carry out all normal activity without restriction |
| 1 | Restricted in strenuous activity but ambulatory and able to carry out light work |
| 2 | Ambulatory and capable of all self-care but unable to carry out any work activities; up and about more than 50% of waking hours |
| 3 | Symptomatic and in a chair or in bed for greater than 50% of the day but not bedridden |
| 4 | Completely disabled; cannot carry out any self-care; totally confined to bed or chair. |

1. Oken MM, Creech RH, Tormey DC, Horton J, Davis TE, McFadden ET, et al. Toxicity and response criteria of the Eastern Cooperative Oncology Group. Am J Clin Oncol. 1982;5(6):649-55.
