# Supplementary material for: The role of day-case thoracoscopy at a district general hospital: A real world observational study
Source: Future Healthc J. 2024 Jul 4;11(3):100158. doi: 10.1016/j.fhj.2024.100158 (PMC11357848; doi:10.1016/j.fhj.2024.100158)
Supplement: Supplementary file 2 [file mmc2.docx]

**Supplementary file 2 – Absolute contraindications to local anesthetic thoracoscopy(1).**

The following listed are absolute contraindications:

-Lung adherent to the chest wall throughout the hemithorax.

-Hypercapnia or severe respiratory distress.

-Uncontrollable cough (making safe entry and movement of thoracoscopes within the chest hazardous).

-Lack of informed consent in a competent patient.

1. Rahman NM, Ali NJ, Brown G, Chapman SJ, Davies RJO, Downer NJ, et al. Local anaesthetic thoracoscopy: British Thoracic Society pleural disease guideline 2010. Thorax. 2010;65(Suppl 2):ii54-ii60.
