# Supplementary material for: The role of day-case thoracoscopy at a district general hospital: A real world observational study
Source: Future Healthc J. 2024 Jul 4;11(3):100158. doi: 10.1016/j.fhj.2024.100158 (PMC11357848; doi:10.1016/j.fhj.2024.100158)
Supplement: Supplementary file 3 [file mmc3.docx]

**DIGITAL SUPPLEMENTARY FILE 3**

EMBASE Search Strategy

15

4 and 10 and 14

18

Advanced

Display Results

More

14

1 or 13

41932

Advanced

Display Results

More

13

thoracoscopic.mp.

31612

Advanced

Display Results

More

12

1 and 10

190

Advanced

Display Results

More

11

1 and 4 and 9 and 10

11

Advanced

Display Results

More

10

5 or 6 or 7 or 8

217871

Advanced

Display Results

More

9

2 or 3

10270

Advanced

Display Results

More

8

ambulatory.mp.

182027

Advanced

Display Results

More

7

same-day.mp.

33453

Advanced

Display Results

More

6

day-case.mp.

6600

Advanced

Display Results

More

5

daycase.mp.

413

Advanced

Display Results

More

4

Malignant pleural effusion.mp. or Pleural Effusion, Malignant/

5260

Advanced

Display Results

More

3

Talc/ or Talc poudrage.mp.

5446

Advanced

Display Results

More

2

Pleurodesis/ or Talc Pleurodesis.mp. or Talc/

10231

Advanced

Display Results

More

1

Thoracoscopy.mp. or Thoracoscopy/

19802

Advanced

Display Results

More

MEDLINE Search Strategy

15

4 and 10 and 14

12

Advanced

Display Results

More

14

1 or 13

22521

Advanced

Display Results

More

13

thoracoscopic.mp.

16567

Advanced

Display Results

More

12

1 and 10

119

Advanced

Display Results

More

11

1 and 4 and 9 and 10

2

Advanced

Display Results

More

10

5 or 6 or 7 or 8

197679

Advanced

Display Results

More

9

2 or 3

3622

Advanced

Display Results

More

8

ambulatory.mp.

178429

Advanced

Display Results

More

7

same-day.mp.

18710

Advanced

Display Results

More

6

day-case.mp.

3628

Advanced

Display Results

More

5

daycase.mp.

120

Advanced

Display Results

More

4

Malignant pleural effusion.mp. or Pleural Effusion, Malignant/

5420

Advanced

Display Results

More

3

Talc/ or Talc poudrage.mp.

2338

Advanced

Display Results

More

2

Pleurodesis/ or Talc Pleurodesis.mp. or Talc/

3568

Advanced

Display Results

More

1

Thoracoscopy.mp. or Thoracoscopy/

11967

Advanced

Display Results

More

Cochrane Library Search Strategy

#1

Thoracoscopy

Limits

783

#2

thoracoscopic

Limits

2284

#3

#1 OR #2

Limits

2657

#4

Malignant pleural effusion

Limits

718

#5

daycase

Limits

705

#6

day-case

Limits

1182

#7

same-day

Limits

3633

#8

ambulatory

Limits

25908

#9

#5 OR #6 OR #7 OR #8

Limits

30019

#10

#3 AND #9

Limits

26
